# AUDIT, AUDIT-C, and AUDIT-3: Drinking Patterns and Screening for Harmful, Hazardous and Dependent Drinking in Katutura, Namibia

**DOI:** 10.1371/journal.pone.0120850

**Published:** 2015-03-23

**Authors:** Puja Seth, Mary Glenshaw, Jennifer H. F. Sabatier, René Adams, Verona Du Preez, Nickolas DeLuca, Naomi Bock

**Affiliations:** 1 Division of Global HIV/AIDS, Centers for Disease Control and Prevention, Atlanta, Georgia, United States of America; 2 Ministry of Health and Social Services, Windhoek, Namibia; 3 Centers for Disease Control and Prevention, Windhoek, Namibia; Kyushu University Faculty of Medical Science, JAPAN

## Abstract

**Objectives:**

To describe alcohol drinking patterns among participants in Katutura, Namibia, and to evaluate brief versions of the AUDIT against the full AUDIT to determine their effectiveness in detecting harmful drinking.

**Methods:**

A cross-sectional survey was conducted in four constituencies and 639 participants, 18 years or older, completed a sociodemographic survey and the AUDIT. The effectiveness of the AUDIT-C (first three questions) and the AUDIT-3 (third question) was compared to the full AUDIT.

**Results:**

Approximately 40% were identified as harmful, hazardous or likely dependent drinkers, with men having a higher likelihood than women (57.2% vs. 31.0%, p<.0001). Approximately 32% reported making and/or selling alcohol from home. The AUDIT-C performed best at a cutoff ≥ 3, better in men (sensitivity: 99.3%, specificity: 77.8%) than women (sensitivity: 91.7%, specificity: 77.4%). The AUDIT-3 performed poorly (maximum sensitivity: < 90%, maximum specificity: <51%). According to AUROC, the AUDIT-C performed better than the AUDIT-3.

**Conclusions:**

A large proportion of participants met criteria for alcohol misuse, indicating a need for screening and referral for further evaluation and intervention. The AUDIT-C was almost as effective as the full AUDIT and may be easier to implement in clinical settings as a routine screening tool in resource-limited settings because of its brevity.

## Background

Alcohol use and abuse are common among people living in sub-Saharan Africa and are characterized by patterns of misuse across many contexts and populations, including social strata, rural and urban environments as well as men and women [1–5]. Among individuals who consume large amounts of alcohol, there is an increase in negative social and personal consequences, including risky behavior, negative health outcomes, disinhibition, sensation seeking, and aggressive/violent behavior [1–3,6–11]. In the 2014 Global Status Report on Alcohol and Health, Namibia was among countries with the highest level of per capita alcohol consumption, particularly on the African continent. When examining both sexes, Namibia also had a higher percentage of persons with alcohol use disorders and alcohol dependence in comparison to the WHO African Region [5]. Even though Namibia is no exception to the high burden of harmful drinking patterns of sub-Saharan Africa, recent studies on drinking patterns and alcohol screening methods in Namibia are limited or have not been conducted. A better understanding of drinking patterns is important to help mitigate the substantial social and health harms associated with heavy drinking.

A study conducted in 2000 by the World Health Organization (WHO) in seven regions found that approximately 50% of men and 25% of women in Namibia reported experiencing at least one alcohol-related problem in the past three months, such as feeling remorse and guilt after drinking or inability to stop drinking [12]. In 2002, the Namibian Ministry of Health and Social Services (MoHSS) found that 56% of Namibian adults were current drinkers and that drinking rates in the capitol, Windhoek, were 70%. Among those who drank, 45% in Windhoek reported recent binge drinking (more than 6 units of alcohol per occasion), and 27% reported the need to consume alcohol in the morning [13]. In 2010, WHO estimated that heavy episodic drinking occurred among 37.2% of the drinking population in Namibia, 15 years and older. Alcohol use disorders (5.1%) and alcohol dependence (3.7%) in Namibia also were markedly higher in comparison to the WHO African region (3.3% and 1.4%, respectively) [5].

Given the estimated high rates of drinking in Namibia, brief and effective alcohol screening tools are necessary to identify individuals with risky drinking patterns in resource-limited settings. The Alcohol Use Disorders Identification Test (AUDIT) is a 10-item, widely used screening instrument developed by WHO. It was designed for use in primary healthcare settings to identify harmful, hazardous or likely dependent drinkers [14–17]. The AUDIT has been widely used in a variety of settings throughout the world, including sub-Saharan Africa [11,18–22]. However, healthcare providers often do not screen for alcohol-related issues, and time constraints may be a contributing factor. Therefore, two abbreviated screening tools have been developed and validated in North America and Europe. The AUDIT-Consumption (AUDIT-C) consists of the first 3 questions of the full 10-item AUDIT, and the AUDIT-3 consists of only the binge drinking question, the third question from the full AUDIT [23].

Evaluations on the sensitivity and specificity of the AUDIT-C and the AUDIT-3 have been conducted among the general population and in various clinical settings. These briefer screening tools distinguish between low risk and high risk drinkers (i.e., harmful, hazardous, or likely dependent). Several studies have found that both the AUDIT-C and AUDIT-3 are comparable to the original AUDIT across various settings and different racial/ethnic groups [24–26]. However, different cutoff scores for high-risk may be needed for men and women on the AUDIT-C, ranging from ≥4 to ≥6 for men and ≥2 to ≥4 for women [26–28]. In settings where the prevalence of alcohol abuse is similar for men and women, the same cutoff score (≥3 to ≥5) could be optimal [27,29]. Overall, the AUDIT-C has been considered comparable to the full AUDIT in countries outside of sub-Saharan Africa.

Although studies have identified the AUDIT-3, which assesses binge drinking (i.e., 6 or more standard drinks in a sitting), as effective in detecting alcohol misuse, the AUDIT-C has performed significantly better than the AUDIT-3 [27,29]. Findings have been less conclusive, particularly among women and when compared to the full AUDIT and the AUDIT-C [25–27].

To our knowledge, no studies have evaluated the effectiveness of the AUDIT-C and AUDIT-3 in sub-Saharan Africa. Recent studies on drinking patterns and alcohol screening methods in Namibia are limited and are needed to help mitigate the substantial social and health harms associated with heavy drinking. For this study, participants were selected from the community of Katutura, which is adjacent to Windhoek. Data on the prevalence of alcohol use and abuse in Katutura do not exist. The community has a substantial number of informal drinking venues (i.e., shebeens), where people make and/or sell alcohol from their home. This is one of the first studies to: [1] describe drinking patterns among adults in four constituencies (political jurisdictions) in Katutura, [2] describe the prevalence of making and selling alcohol from home, and [3] compare the AUDIT-C and AUDIT-3 against the full 10-item AUDIT in their effectiveness to detect high-risk drinking (i.e. harmful, hazardous or likely dependent) among this population.

## Methods

### Study Design and Participants

This study was a population-based, cross-sectional survey of adults living in randomly selected households in a convenience sample of four constituencies in Katutura. The four constituencies included: Tobias Hainyeko, Moses Gareb, Samora Machel, and Soweto. Many neighborhoods are informal settlements. These constituencies were selected because a community-based HIV prevention and care outreach program targeting high risk communities had systematically mapped all households (20,863) in 2008, providing a recent and accurate sampling frame. Households were chosen using a 3-stage stratified sampling design. Each stratum was further sub-stratified into 50 distinct geographic areas. Finally, a proportional number of houses were randomly selected within each geographic area. Participant inclusion criteria were 18 years or older, verbal informed consent, and the ability to answer questions in English, Oshiwambo, or Afrikaans. The protocol, including consent procedures, was approved by the Namibian MoHSS and the Institutional Review Board at the Centers for Disease Control and Prevention prior to implementation.

### Procedures

Each person approached in the selected households was asked to identify a private place in the home or outdoors to administer the survey. The survey administrator then explained the purpose of the survey, including the AUDIT screen and supplementary questions, the interpretation of results, and resources that would be provided should alcohol misuse be identified. The administrator assured confidentiality. For those willing to participate, verbal but not written consent was obtained and recorded on the screening form to assure anonymity, and the survey was administered individually. Participants were free to not answer any questions that made them uncomfortable and to stop the interview at any time.

### Measures

Sociodemographic variables included sex, age, and paid employment. All participants were asked whether they made or sold alcohol from their home. Those who acknowledged drinking alcohol in the last year were asked if they ever drank home-brewed beer, what type of alcohol they drank most of the time (i.e., homebrewed beer, bottled beer, wine, or spirits), and where they typically purchased alcohol.

#### Alcohol Use Disorders Identification Test (AUDIT)

The AUDIT is a widely used 10-item screening measure that assesses alcohol use during the previous twelve months. While not intended to be clinically diagnostic, the AUDIT indicates the presence and severity of an alcohol problem or alcohol use disorder [14,16]. AUDIT responses are scored on a 3-point and 5-point Likert scale and total scores range from 0–40. Questions cover a variety of domains regarding drinking behavior: harmful use (guilt, unconsciousness, injury), hazardous alcohol use (frequency, quantity, heavy drinking), and dependence indications (reduced control, conspicuousness, morning consumption). Based on the total score, participants are categorized into five categories: Non-drinker (score: 0), low-risk drinker (scores: 1–7), harmful drinker (scores: 8–15), hazardous drinker (scores: 16–19), or likely dependent on alcohol (scores: ≥20) [14]. As mentioned previously, the AUDIT-C consists of the first 3 questions, and the AUDIT-3 consists of the third question (binge drinking) [23].

### Data Analyses

Primary analyses described patterns of alcohol consumption among residents of Katutura and examined sex differences. These patterns were analyzed, using survey software, accounting for sample weights, clustering (household), and stratification (constituency), and estimates were population-based, representing the population of the four constituencies of Katutura. Analyses were generated from R 2.15.1 [30,31] and SAS 9.3.

Additional analyses compared the AUDIT-3 and AUDIT-C for sensitivity and specificity against the full AUDIT cut-off score ≥ 8 for the overall sample and for men and women separately. Sensitivity, specificity, and 95% confidence intervals account for the sampling design. Sensitivity refers to the “true positive rate,” which measures the proportion of persons who are correctly identified as high-risk drinkers. Specificity refers to the “true negative rate,” which measures the proportion of persons who are correctly identified as not being high-risk drinkers. The analyses illustrated which cut-off (s) for the AUDIT-3 or AUDIT-C most closely predicted harmful, hazardous, or likely dependent drinking, as defined previously for the original AUDIT. In addition, the area under the receiver-operating curve (AUROC) was used, which compared the areas under the curves with a distribution-free permutation procedure for curves based on data from a paired design [32]. With AUROC, larger areas indicate superior performance, with 1 indicating perfect performance. The anonymized dataset is available upon request from the analysis working group, comprising the corresponding and senior authors, members of the MOHSS, and CDC.

## Results

In March 2009, 85% (340) of the 400 approached households participated, which represented 1.6% of households registered across all 4 constituencies (20,863). A total of 639 adults participated; the median number of surveys per household was two, and the range was one to seven. Approximately two-thirds of participants were women (64.6%), and there were no significant differences by sex in the age distribution (p = .57) (Table 1). Participants’ ages ranged from 18 to 80 years, with the largest proportion (43.5%) being 20–29 years. Approximately half (51.3%) reported paid employment, with no significant differences by sex (p = .79).

**Table 1:** Demographic Characteristics*.

	Men				Women				Overall				
	n	%	95% CI		n	%	95% CI		n	%	95% CI		p-value
**Overall**	222	35.4%	(31.7%	, 39.0%)	417	64.6%	(60.7%	, 68.0%)	639	100%			
**Age**													
**Mean**		30.4	(28.8	, 32.1)		30.5	(29.4	, 31.6)		30.1	(29.3	, 30.9)	0.9506
18–20	26	12.3%	(7.4%	, 17.1%)	53	13.7%	(9.8%	, 17.6%)	79	12.2%	(9.6%	, 14.7%)	0.5693
>20–29	103	44.0%	(36.3%	, 51.7%)	178	38.4%	(33.2%	, 43.7%)	281	43.5%	(39.6%	, 47.3%)	
>29–39	51	21.4%	(15.2%	, 27.6%)	118	27.9%	(23.2%	, 32.6%)	169	25.8%	(22.5%	, 29.2%)	
>39	32	18.7%	(11.7%	, 25.7%)	55	16.8%	(12.4%	, 21.3%)	87	14.8%	(12.2%	, 17.5%)	
Missing	10	3.7%	(1.3%	, 6.1%)	13	3.2%	(1.3%	, 5.0%)	23	3.7%	(2.2%	, 5.2%)	
**Employment status**													
Employed (any capacity)	115	49.3%	(41.0%	, 57.5%)	211	48.2%	(42.8%	, 53.6%)	326	51.3%	(47.5%	, 55.2%)	0.7919
Unemployed	104	49.2%	(41.0%	, 57.5%)	202	50.9%	(45.5%	, 56.3%)	306	47.7%	(43.8%	, 51.5%)	
Missing	3	1.5%	-(0.4%	, 3.3%)	4	0.8%	(0.0%	, 1.7%)	7	1.0%	(0.3%	, 1.7%)	
**Makes or sell alcohol from home**													
No	151	73.8%	(67.6%	, 79.9%)	263	63.7%	(58.5%	, 68.9%)	414	67.0%	(63.0%	, 70.9%)	0.0548
Yes	69	25.5%	(19.4%	, 31.7%)	150	35.3%	(30.2%	, 40.5%)	219	32.2%	(28.2%	, 36.1%)	
Missing	2	0.7%	-(0.3%	, 1.7%)	4	0.9%	(0.0%	, 2.0%)	6	0.8%	(0.0%	, 1.7%)	
**Drinks homebrewed beer** **													
No	51	28.6%	(21.1%	, 36.2%)	59	21.4%	(15.9%	, 26.8%)	110	24.0%	(19.7%	, 28.3%)	0.0595
Yes	123	67.4%	(59.4%	, 75.5%)	223	77.3%	(71.8%	, 82.8%)	346	73.7%	(69.3%	, 78.2%)	
Missing	5	3.9%	(0.0%	, 8.3%)	6	1.3%	(0.2%	, 2.4%)	11	2.3%	(0.6%	, 3.9%)	
**Type of alcohol most frequently consumed** **													
Homebrewed beer	61	36.3%	(27.5%	, 45.0%)	154	54.7%	(48.2%	, 61.3%)	215	48.0%	(42.9%	, 53.1%)	0.0019
Bottled beer	91	50.3%	(41.5%	, 59.1%)	109	38.1%	(31.7%	, 44.5%)	200	42.6%	(37.5%	, 47.7%)	
Wine	15	6.6%	(2.6%	, 10.6%)	14	4.2%	(1.7%	, 6.7%)	29	5.1%	(3.0%	, 7.2%)	
Spirits	8	4.8%	(1.0%	, 8.7%)	4	0.9%	(0.0%	, 1.8%)	12	2.3%	(0.8%	, 3.9%)	
Missing	4	2.0%	(0.0%	, 4.0%)	7	2.0%	(0.3%	, 3.8%)	11	2.0%	(0.7%	, 3.3%)	
**Location of typical alcohol purchases** **													
Neighborhood Shebeen	155	85.5%	(79.5%	, 91.5%)	262	91.2%	(87.5%	, 94.9%)	417	89.1%	(86.0%	, 92.3%)	0.1231
Commercial Bar	8	6.8%	(2.0%	, 11.6%)	6	2.3%	(0.2%	, 4.4%)	14	4.0%	(1.7%	, 6.2%)	
Bottle Store	12	5.8%	(2.3%	, 9.2%)	11	3.8%	(1.4%	, 6.2%)	23	4.5%	(2.5%	, 6.5%)	
Missing	4	1.9%	(0.0%	, 3.9%)	9	2.7%	(0.7%	, 4.6%)	13	2.4%	(1.0%	, 3.8%)	

*These statistics are population estimates, which imply stratification by constituency and weighting by household

**These questions were only asked of those participants who reported being current drinkers (i.e., AUDIT score ≥ 1 on AUDIT question #1).

Approximately one-third (32.2%) of participants reported making or selling alcohol from their homes, including a mix of homebrewed and manufactured alcohol products (Table 1). Among the 467 (73.1%) self-reported current drinkers (i.e., consumed alcohol in the past 12 months), consumption of homebrewed (48.0%) and bottled beer (42.6%) was more prevalent than wine (5.1%) or spirits (2.3%). A significantly higher proportion of men reported consumption of bottled beer than women (50.3% vs. 38.1%) and a higher proportion of women reported consumption of homebrewed beer (54.7% vs. 36.3%) (p = 0.0019).

### AUDIT—Drinking Patterns in Katutura

Following AUDIT guidelines, if respondents answered “never” to the first AUDIT question (“How often in the past year did you have a drink containing alcohol?”), then questions 2 through 8 were skipped. AUDIT scores were obtained on 625 participants because of missing data on key questions for 14 participants. The first question was completed by 100% (639) of participants, and 26.9% (172) responded “never.” Thus, 73.1% indicated alcohol consumption in the previous 12 months, and the denominator for questions 2 through 8 was 467. In terms of data completeness, the AUDIT-3 was completed by all 467 current drinkers and the AUDIT-C by 466.

Table 2 displays participants’ drinking patterns and alcohol risk categories. The average full AUDIT score was 7.2 (95% CI: 6.5, 7.9). There were sex differences, with a mean score of 9.5 (95% CI: 8.4, 10.6) for men and 6.1 (95% CI: 5.3, 6.9) for women (p<.0001). When examining drinking categories, 39.5% (95% CI: 35.1%, 43.9%) were classified as harmful, hazardous or likely dependent drinkers by the full AUDIT (AUDIT ≥ 8). The breakdown by sex revealed that 57.2% (95% CI: 49.6%, 64.8%) of men, and 31% (95% CI: 26.1%, 36%) of women were categorized as harmful, hazardous drinkers or likely dependent on alcohol (p<.0001).

**Table 2:** AUDIT Survey by Sex*.

AUDIT Question	Men				Women				Overall				p-value
	n	%	95% Confidence Interval		n	%	95% Confidence Interval		n	%	95% Confidence Interval		
**1. How often do you have a drink containing alcohol?** ** ^,^ ^†^													0.0002
Never	43	20.2%	(14.2%	, 26.3%)	129	33.5%	(28.2%	, 38.7%)	172	29.2%	(25.1%	, 33.3%)	
Monthly or less	53	26.3%	(18.8%	, 33.7%)	128	31.5%	(26.4%	, 36.7%)	181	29.8%	(25.6%	, 34.0%)	
2–4 times per month	42	19.6%	(13.3%	, 25.8%)	66	15.1%	(11.3%	, 18.9%)	108	16.5%	(13.2%	, 19.8%)	
2–3 times per week	40	18.8%	(11.9%	, 25.7%)	37	7.2%	(4.6%	, 9.8%)	77	10.9%	(8.0%	, 13.9%)	
4+ times per week	44	15.2%	(10.2%	, 20.2%)	57	12.7%	(9.2%	, 16.3%)	101	13.5%	(10.7%	, 16.4%)	
**2. How many standard drinks do you have on a typical day when you are drinking?** ** ^,^ ^†^													0.0075
1–2 drinks	28	16.1%	(9.8%	, 22.4%)	81	31.2%	(25.0%	, 37.4%)	109	25.7%	(21.0%	, 30.4%)	
3–4 drinks	54	31.8%	(22.6%	, 41.0%)	105	35.0%	(29.0%	, 41.1%)	159	33.8%	(28.8%	, 38.9%)	
5–6 drinks	43	25.6%	(17.1%	, 34.1%)	46	15.0%	(10.4%	, 19.6%)	89	18.9%	(14.5%	, 23.2%)	
7–9 drinks	18	11.6%	(5.6%	, 17.5%)	26	8.3%	(4.8%	, 11.8%)	44	9.5%	(6.4%	, 12.5%)	
10 or more drinks	36	14.9%	(9.5%	, 20.3%)	29	10.3%	(6.3%	, 14.2%)	65	12.0%	(8.8%	, 15.1%)	
Missing					1	0.2%	(0.0%	, 0.6%)	1	0.1%	(0.0%	, 0.4%)	
**3. How often do you have six or more standard drinks on one occasion?** ** ^,^ *** ^,^ ^†^													0.0024
Never	39	25.2%	(17.1%	, 33.4%)	116	42.9%	(36.4%	, 49.4%)	155	36.5%	(31.3%	, 41.6%)	
Less than monthly	40	20.9%	(13.2%	, 28.5%)	77	24.6%	(19.2%	, 30.0%)	117	23.2%	(18.9%	, 27.6%)	
Monthly	49	29.5%	(20.7%	, 38.4%)	46	16.4%	(11.5%	, 21.3%)	95	21.2%	(16.6%	, 25.7%)	
Weekly	29	13.6%	(8.3%	, 18.9%)	29	9.2%	(5.6%	, 12.8%)	58	10.8%	(7.8%	, 13.8%)	
Daily or almost daily	22	10.8%	(5.6%	, 16.0%)	20	6.9%	(3.7%	, 10.0%)	42	8.3%	(5.5%	, 11.0%)	
**4. How often during the last year have you found that you were not able to stop drinking once you had started?** ^†^													0.3765
Never	88	52.1%	(42.7%	, 61.4%)	174	61.7%	(55.4%	, 68.0%)	262	58.2%	(53.0%	, 63.4%)	
Less than monthly	35	17.7%	(11.3%	, 24.1%)	53	17.6%	(12.6%	, 22.6%)	88	17.7%	(13.7%	, 21.6%)	
Monthly	31	18.3%	(10.2%	, 26.4%)	31	11.7%	(7.2%	, 16.2%)	62	14.1%	(9.9%	, 18.3%)	
Weekly	14	7.0%	(2.8%	, 11.2%)	15	5.1%	(2.3%	, 8.0%)	29	5.8%	(3.5%	, 8.2%)	
Daily or almost daily	9	4.4%	(0.9%	, 7.8%)	14	3.7%	(1.5%	, 5.8%)	23	3.9%	(2.1%	, 5.8%)	
Missing	2	0.6%	(0.0%	, 1.3%)	1	0.2%	(0.0%	, 0.5%)	3	0.3%	(0.0%	, 0.7%)	
**5. How often during the last year have you failed to do what was normally expected of you because of drinking?** ^†^													0.1408
Never	92	51.2%	(41.7%	, 60.7%)	185	65.2%	(59.0%	, 71.4%)	277	60.1%	(54.8%	, 65.4%)	
Less than monthly	46	27.3%	(19.0%	, 35.6%)	59	20.1%	(14.7%	, 25.5%)	105	22.7%	(18.1%	, 27.3%)	
Monthly	16	7.2%	(3.5%	, 10.9%)	19	6.4%	(3.1%	, 9.8%)	35	6.7%	(4.2%	, 9.2%)	
Weekly	15	8.7%	(2.1%	, 15.2%)	15	4.6%	(1.9%	, 7.2%)	30	6.1%	(3.1%	, 9.1%)	
Daily or almost daily	10	5.6%	(1.9%	, 9.3%)	9	3.4%	(1.0%	, 5.8%)	19	4.2%	(2.2%	, 6.3%)	
Missing					1	0.3%	(0.0%	, 0.8%)	1	0.2%	(0.0%	, 0.5%)	
**6. How often during the last year have you needed a first drink in the morning to get yourself going after a heavy drinking session?** ^†^													0.3420
Never	115	63.3%	(54.2%	, 72.4%)	197	70.0%	(64.0%	, 76.0%)	312	67.6%	(62.5%	, 72.6%)	
Less than monthly	26	15.0%	(7.8%	, 22.3%)	35	11.0%	(7.0%	, 14.9%)	61	12.4%	(8.8%	, 16.1%)	
Monthly	18	10.0%	(4.9%	, 15.1%)	23	7.6%	(4.2%	, 11.0%)	41	8.5%	(5.7%	, 11.3%)	
Weekly	15	8.2%	(3.2%	, 13.1%)	12	4.7%	(1.9%	, 7.5%)	27	6.0%	(3.4%	, 8.5%)	
Daily or almost daily	5	3.5%	(0.0%	, 7.1%)	17	5.0%	(2.4%	, 7.7%)	22	4.5%	(2.4%	, 6.6%)	
Missing					4	1.7%	(0.0%	, 3.6%)	4	1.1%	(0.0%	, 2.3%)	
**7. How often during the last year have you had a feeling of guilt or remorse after drinking?** ^†^													0.0861
Never	74	43.3%	(34.3%	, 52.3%)	150	52.2%	(45.7%	, 58.7%)	224	48.9%	(43.9%	, 54.0%)	
Less than monthly	49	25.0%	(17.5%	, 32.5%)	74	24.1%	(18.4%	, 29.8%)	123	24.4%	(20.0%	, 28.9%)	
Monthly	29	15.5%	(9.6%	, 21.4%)	26	10.3%	(5.8%	, 14.8%)	55	12.2%	(8.7%	, 15.8%)	
Weekly	15	9.0%	(3.8%	, 14.2%)	12	3.6%	(1.3%	, 5.8%)	27	5.5%	(3.2%	, 7.9%)	
Daily or almost daily	10	5.8%	(0.9%	, 10.8%)	25	9.5%	(5.6%	, 13.5%)	35	8.2%	(5.1%	, 11.2%)	
Missing	2	1.3%	(0.0%	, 3.3%)	1	0.3%	(0.0%	, 0.9%)	3	0.7%	(0.0%	, 1.5%)	
**8. How often during the last year have you been unable to remember what happened the night before because you had been drinking?** ^†^													0.1169
Never	169	79.1%	(73.3%	, 85.0%)	345	84.1%	(80.1%	, 88.0%)	514	82.5%	(79.2%	, 85.7%)	
Less than monthly	23	7.4%	(3.8%	, 11.0%)	39	8.1%	(5.1%	, 11.0%)	62	7.9%	(5.5%	, 10.2%)	
Monthly	12	5.5%	(1.9%	, 9.0%)	16	3.8%	(1.7%	, 5.8%)	28	4.3%	(2.5%	, 6.1%)	
Weekly	9	4.7%	(1.2%	, 8.1%)	6	1.1%	(0.2%	, 2.1%)	15	2.3%	(1.0%	, 3.6%)	
Daily or almost daily	9	3.3%	(1.0%	, 5.6%)	10	2.8%	(0.9%	, 4.8%)	19	3.0%	(1.5%	, 4.5%)	
Missing					1	0.1%	(0.0%	, 0.4%)	1	0.1%	(0.0%	, 0.3%)	
**9. Have you or someone else been injured because of your drinking?**													0.0080
Never	172	77.8%	(70.5%	, 85.1%)	362	88.6%	(85.4%	, 91.9%)	534	85.1%	(81.8%	, 88.4%)	
Yes, during last year	29	13.0%	(6.7%	, 19.4%)	27	5.8%	(3.4%	, 8.3%)	56	8.2%	(5.5%	, 10.9%)	
Yes, not in the last year	20	9.0%	(4.6%	, 13.3%)	26	5.2%	(3.0%	, 7.5%)	46	6.4%	(4.4%	, 8.5%)	
Missing	1	0.2%	(0.0%	, 0.7%)	2	0.3%	(0.0%	, 0.7%)	3	0.3%	(0.0%	, 0.6%)	
**10. Has a relative, friend, doctor, or other healthcare worker been concerned about your drinking or suggested you cut down?**													0.0028
Never	140	64.7%	(56.8%	, 72.6%)	323	78.9%	(74.4%	, 83.3%)	463	74.3%	(70.3%	, 78.3%)	
Yes, during last year	36	18.1%	(11.1%	, 25.1%)	38	8.8%	(5.8%	, 11.8%)	74	11.8%	(8.7%	, 14.9%)	
Yes, not in the last year	46	17.2%	(12.0%	, 22.5%)	56	12.3%	(8.7%	, 15.9%)	102	13.9%	(11.0%	, 16.8%)	
**Total AUDIT Score (mean)**		9.5	(8.4	, 10.6)		6.1	(5.3	, 6.9)		7.2	(6.5	, 7.9)	<0.0001
**Alcohol Risk Groupings**													
Not at Risk (< = 7)	86	40.3%	(32.8%	, 47.8%)	272	67.5%	(62.5%	, 72.5%)	359	56.6%	(52.8%	, 60.4%)	<0.0001
Harmful, Hazardous, or Likely Dependent (> = 8)	130	57.2%	(49.6%	, 64.8%)	136	31.0%	(26.1%	, 36.0%)	266	39.5%	(35.1%	, 43.9%)	
Harmful (8–15)	83	38.3%	(30.6%	, 46.0%)	86	19.7%	(15.3%	, 24.0%)	168	26.3%	(22.8%	, 29.8%)	
Hazardous (16–19)	18	7.1%	(3.4%	, 10.7%)	15	3.3%	(1.4%	, 5.2%)	33	5.0%	(3.3%	, 6.7%)	
Likely Dependent (> = 20)	30	12.5%	(7.8%	, 17.3%)	35	8.1%	(5.1%	, 11.0%)	65	10.1%	(7.8%	, 12.4%)	
Missing	5	1.7%	(0.0%	, 3.4%)	9	1.5%	(0.5%	, 2.5%)	14	2.0%	(1.0%	, 3.0%)	

*These statistics are population estimates, which imply stratification by constituency and weighting by household.

** Questions 1–3 represents the AUDIT-C.

*** Question 3 represents the AUDIT-3.

^†^ Time period for these questions is during the previous 12 months.

Among current drinkers, 63.5% (95% CI: 58.4%, 68.7%) reported binge drinking at least once in the previous year, which included 74.8% (95% CI: 66.6%, 82.9%) of men and 57.1% (95% CI: 50.6%, 63.6%) of women. Although significantly higher among men, frequent binge drinking was high among both sexes; 53.2% of men and 32.5% of women reported binge drinking on a monthly, weekly or daily basis and 10.8% of men and 6.9% of women reported binge drinking daily or almost daily (Table 2).

### AUDIT-C and AUDIT-3

Tables 3 and 4 show how the AUDIT-C and AUDIT-3, respectively, compared with the full AUDIT at classifying harmful, hazardous, and likely dependent drinkers (i.e. high risk drinking). Overall, the highest sensitivity with a specificity of at least 70% for the AUDIT-C was a score ≥3 (Table 3), where sensitivity was 95.2% (95% CI: 91.9%, 98.6%), and specificity was 77.5% (95% CI: 72.5%, 82.5%). Sensitivity was higher for men (99.3%, 95% CI: 98.3%, 100%) than for women (91.7%, 95% CI: 85.5%, 97.8%), while specificity was similar (men: 77.8%, 95% CI: 68.4%, 87.3%; women: 77.4%, 95% CI: 71.7%, 83.2%).

**Table 3:** Sensitivity and Specificity of Cut-offs for AUDIT-C to the full AUDIT*.

	Sensitivity		Specificity	
	%	95% Confidence Interval	%	95% Confidence Interval
**AUDIT-C > = 1**				
Men	100.0	(100.0, 100.0)	49.3	(37.2, 61.3)
Women	99.7	(99.2, 100.0)	49.5	(42.5, 56.5)
Overall	99.8	(99.6, 100.0)	49.4	(43.3, 55.6)
**AUDIT-C > = 2**				
Men	100.0	(100.0, 100.0)	63.5	(51.9, 75.1)
Women	96.1	(91.9, 100.0)	65.2	(58.6, 71.9)
Overall	97.9	(95.7, 100.0)	64.8	(59.0, 70.6)
**AUDIT-C > = 3**				
Men	99.3	(98.3, 100.0)	77.8	(68.4, 87.3)
Women	91.7	(85.5, 97.8)	77.4	(71.7, 83.2)
Overall	95.2	(91.9, 98.6)	77.5	(72.5, 82.5)
**AUDIT-C > = 4**				
Men	91.5	(84.9, 98.1)	88.8	(82.2, 95.3)
Women	88.4	(81.5, 95.4)	86.6	(81.8, 91.5)
Overall	89.9	(85.1, 94.7)	87.1	(83.1, 91.1)
**AUDIT-C > = 5**				
Men	81.9	(71.2, 92.6)	94.9	(90.7, 99.1)
Women	77.4	(68.6, 86.2)	93.5	(90.2, 96.8)
Overall	79.5	(72.9, 86.2)	93.8	(91.1, 96.5)
**AUDIT-C > = 6**				
Men	68.8	(56.8, 80.7)	99.5	(98.5, 100.0)
Women	60.5	(50.7, 70.2)	98.1	(96.1, 100.0)
Overall	64.3	(56.6, 72.1)	98.4	(96.9, 99.9)
**AUDIT-C > = 7**				
Men	52.0	(41.5, 62.5)	99.5	(98.5, 100.0)
Women	43.4	(33.7, 53.1)	100.0	(100.0, 100.0)
Overall	47.4	(40.3, 54.6)	99.9	(99.7, 100.0)
**AUDIT-C > = 8**				
Men	38.4	(28.6, 48.2)	100.0	(100.0, 100.0)
Women	30.4	(21.6, 39.2)	100.0	(100.0, 100.0)
Overall	34.2	(27.5, 40.8)	100.0	(100.0, 100.0)
**AUDIT-C > = 9**				
Men	31.8	(22.4, 41.2)	100.0	(100.0, 100.0)
Women	24.4	(16.1, 32.6)	100.0	(100.0, 100.0)
Overall	27.8	(21.6, 34.1)	100.0	(100.0, 100.0)
**AUDIT-C > = 10**				
Men	11.9	(6.0, 17.9)	100.0	(100.0, 100.0)
Women	15.1	(8.0, 22.2)	100.0	(100.0, 100.0)
Overall	13.6	(8.9, 18.3)	100.0	(100.0, 100.0)
**AUDIT-C > = 11**				
Men	9.8	(4.1, 15.5)	100.0	(100.0, 100.0)
Women	11.7	(5.4, 18.0)	100.0	(100.0, 100.0)
Overall	10.8	(6.6, 15.1)	100.0	(100.0, 100.0)
**AUDIT-C > = 12**				
Men	3.6	(1.0, 6.2)	100.0	(100.0, 100.0)
Women	7.3	(1.9, 12.6)	100.0	(100.0, 100.0)
Overall	5.5	(2.3, 8.7)	100.0	(100.0, 100.0)

*These statistics are population estimates, which imply stratification by constituency and weighting by household.

**Table 4:** Sensitivity and Specificity of Cut-offs for AUDIT-3 to the full AUDIT*.

	Sensitivity				Specificity		
	%		95% Confidence Interval		%		95% Confidence Interval
**AUDIT-3 > = 1**							
Men	86.0	(77.6, 94.3)			29.4	(17.8, 41.1)	
Women	84.8	(76.9, 92.6)			35.4	(29.1, 41.8)	
Overall	85.3	(79.7, 91.0)			34.1	(28.4, 39.7)	
**AUDIT-3 > = 2**							
Men	69.8	(58.4, 81.3)			46.2	(33.7, 58.7)	
Women	62.4	(52.6, 72.3)			48.6	(41.6, 55.5)	
Overall	65.9	(58.4, 73.4)			48.0	(41.9, 54.1)	
**AUDIT-3 > = 3**							
Men	34.0	(24.7, 43.3)			50.7	(38.7, 62.8)	
Women	33.8	(24.4, 43.2)			50.5	(43.5, 57.5)	
Overall	33.9	(27.2, 40.6)			50.6	(44.4, 56.7)	
**AUDIT-3 > = 4**							
Men	15.1	(7.8		, 22.3)	50.7	(38.7, 62.8)	
Women	14.7	(7.8		, 21.6)	50.5	(43.5, 57.5)	
Overall	14.9	(9.8		, 20.0)	50.6	(44.4, 56.7)	

*These statistics are population estimates, which imply stratification by constituency and weighting by household.

For the AUDIT-3 (Table 4), a score ≥1 obtained the highest sensitivity (above 80%), but specificity scores were poor. The sensitivity for the overall sample was 85.3% (95% CI: 79.7, 91.0), and specificity was 34.1% (95% CI: 28.4%, 39.7%). Specificity was lower for men (29.4%, 95% CI: 17.8%, 41.1%) than for women (35.4%, 95% CI: 29.1%, 41.8%), whereas sensitivity was similar (men: 86%, 95% CI: 77.6%, 94.3%; women: 84.8%, 95% CI: 76.9%, 92.6%).

**Fig 1:**
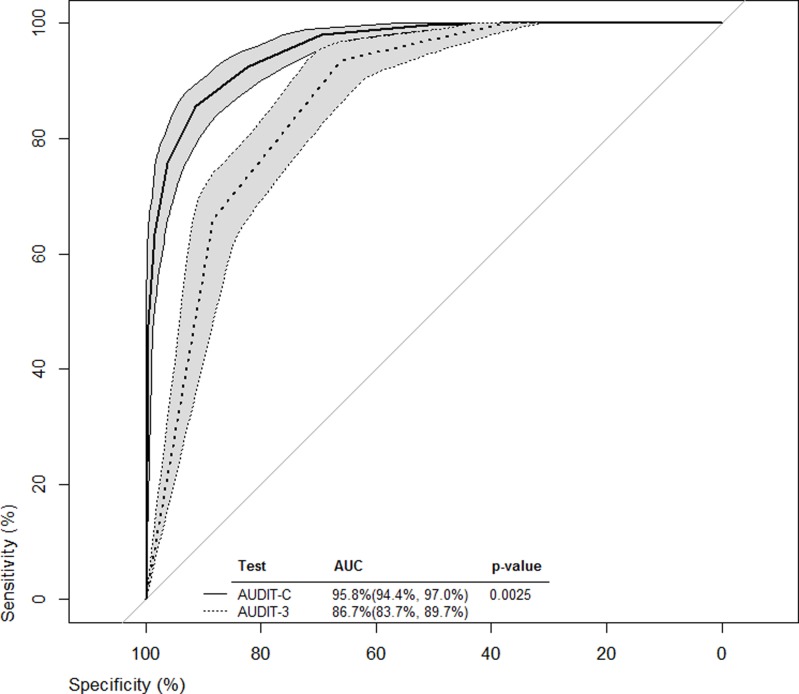
ROC curves for the overall sample from all cut-off values for both AUDIT-C and AUDIT-3.

**Fig 2:**
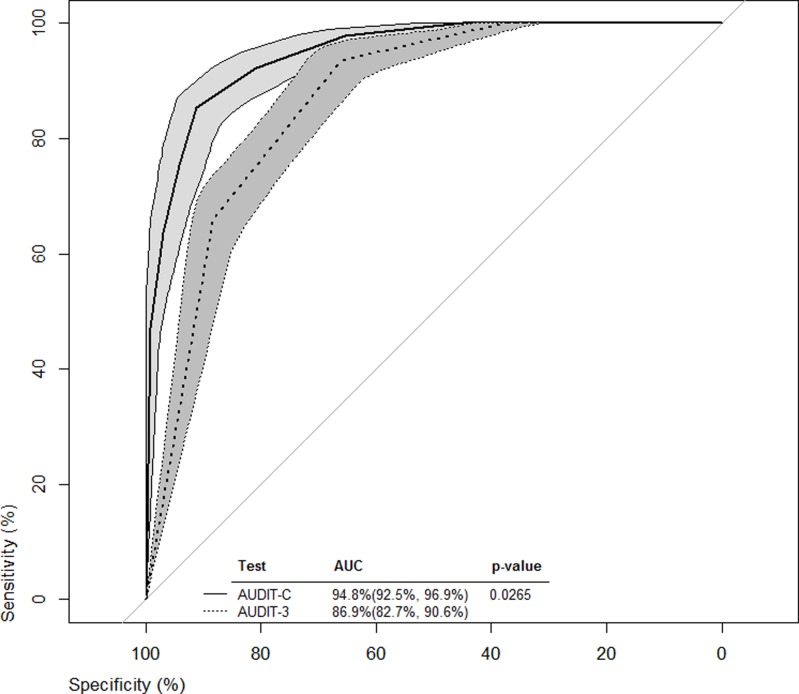
ROC curves for women from all cut-off values for both AUDIT-C and AUDIT-3.

**Fig 3:**
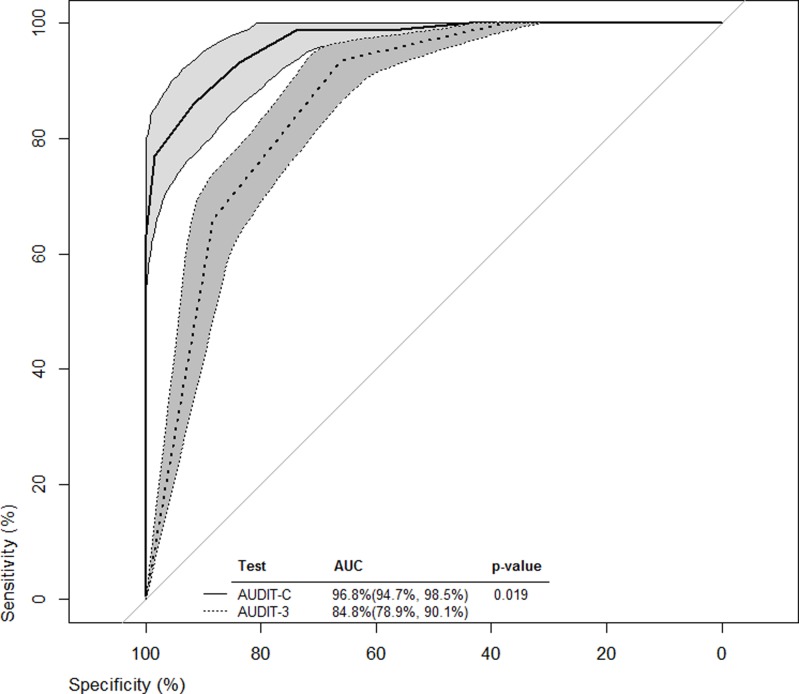
ROC curves for men from all cut-off values for both AUDIT-C and AUDIT-3.

ROC curves from all cut-off values were constructed for both the AUDIT-C and AUDIT-3 (Figs. 1–3). Bootstrapped 95% confidence intervals for sensitivity at given specificity points, provided by the analysis in Tables 3 and 4 and vice versa, were overlaid on the ROC curves to provide some estimate of the precision of this analyses. For example, with the AUDIT-C (Fig. 1, solid-lined curve) and a sensitivity of 95.2% (Table 3, cutoff score = 3), the bootstrapped 95% confidence interval for specificity around the ROC curve was estimated to be 69.6%- 83.3%, which was wider than the 95% confidence interval estimated using methods for complex samples.

The AUROC illustrated the ability of the AUDIT-C and AUDIT-3 to classify levels of alcohol consumption risk when compared to the full AUDIT. Both AUROCs for the briefer screening tools were significantly superior to the line of identity (x-axis = 1—y-axis) at classifying harmful, hazardous, or likely dependent drinkers. However, for the overall sample and for each sex, the AUROCs indicated that AUDIT-C performed significantly better than the AUDIT-3 in classifying harmful, hazardous, or likely dependent drinkers.

## Discussion

This is one of the first studies conducted on drinking patterns and alcohol screening methods in Katutura, Namibia. This population-based study examined alcohol use and abuse patterns among household participants in four constituencies in Katutura. Overall, rates for both were alarming. Over 70% of participants self-identified as current drinkers, with nearly 40% of those reporting harmful, hazardous or likely dependent drinking, and 63.5% reporting binge drinking in the past 12 months. These findings are similar to the study conducted by the Namibian Ministry of Health and Social Services approximately 10 years earlier [13], indicating that harmful drinking behavior may not have changed in Namibia in over a decade. Additionally, although rates were alarming for both men and women, men were more likely to report harmful, hazardous, or especially, likely dependent drinking behaviors than women. Specifically, daily, weekly and monthly binge drinking rates were significantly higher for men.

This study also is the first to systematically document the prevalence of informal alcohol sales in Katutura. The finding that 32.2% reported making or selling alcohol from their homes indicates a lack of enforcement of national laws governing alcohol sales in Namibia. Both men and women reported making or selling alcohol from their homes, but rates were higher among women (35.3% vs. 25.5%). Further, consumption of homebrewed beer was highly prevalent, particularly among women. Consumption of informally manufactured alcohol complicates accurate assessment of alcohol use and alcohol interventions for high-risk drinking. Further research is needed to address the complexities of alcohol consumption and screening among this population.

Finally, to our knowledge, this study is the first to evaluate the effectiveness of the AUDIT-C and AUDIT-3 in detecting harmful, hazardous or likely dependent drinking in comparison to the full 10-item AUDIT in Namibia. In this resource limited setting, the AUDIT-3 performed poorly. However, the AUDIT-C performed better than the AUDIT-3 among the overall sample and among both sexes, which is consistent with previous findings from other settings and countries [27,29]. Therefore, the brief 3-item version of the AUDIT (AUDIT-C) may be effective in detecting high-risk drinking, whereas the 1-item version (AUDIT-3) may not be as effective. At a cut-off score of ≥ 3, essentially all men (99.3%) identified from the full AUDIT as high-risk drinkers would be identified with the AUDIT-C, although some women may be missed as sensitivity was 91.7%. As with the full AUDIT, further clinical assessment of those who screen positive for high-risk drinking is necessary to confirm the finding.

There are limitations to this study. The participants consisted of adults present at the time of a single household visit. For safety reasons, data were collected only during daylight hours and household members working may have been missed. Therefore, more women are represented than men. Previous research has indicated that alcohol use levels may be higher in nonworking populations; therefore, alcohol consumption rates among men may be skewed and overestimated. Other limitations are related to the screening tools. The abbreviated versions of the AUDIT were compared only with the full AUDIT and were not administered independently of the full 10-question AUDIT. Results for the briefer versions may have been different, if administered separately. Since 48% of participants reported primary consumption of homebrewed rather than manufactured alcohol, assessment of alcohol content is difficult. The common practice of sharing drinks among a group and drinking from a variety of non-standard containers presents a challenge in quantifying alcohol consumption among this population. Finally, this study was conducted in one community, Katutura, where sociodemographic characteristics may not be similar to neighboring communities or other parts of Namibia. Therefore, results may not be generalizable, and further research with diverse geographic populations is needed.

Many factors limit alcohol screening in both clinic and community settings, including social acceptability or lack thereof regarding alcohol use, limited time of providers, and lack of training [33,34]. However, given that alcohol is associated with high-risk sexual behavior and negative health and social outcomes, it is important for providers in both settings to routinely screen patients for current alcohol use and provide alcohol reduction counselling to those who report harmful, hazardous or likely dependent drinking. Previous research has indicated that healthcare settings are an avenue for brief, effective interventions for alcohol issues [35,36]. For certain populations (e.g., HIV-positive persons, pregnant women), screening and intervention should be offered if any alcohol use is reported. The current findings indicate that providers in resource limited settings may use the AUDIT-C as a very brief, effective alcohol screening tool to identify persons who need further evaluation and intervention. Despite legislation and policies regulating alcohol sales in Namibia, the relatively large prevalence of high-risk drinking and consumption of homebrewed in Namibia indicates potential barriers in enforcing these policies. Programs to promote alternative income generation and enforcement of alcohol sales restrictions should be considered. Additional research and guidance also are needed to help curb high-risk drinking in the context of these unique challenges.
